# Regulation of Oligodendrocyte Functions: Targeting Lipid Metabolism and Extracellular Matrix for Myelin Repair

**DOI:** 10.3390/jcm9020470

**Published:** 2020-02-08

**Authors:** Davide Marangon, Marta Boccazzi, Davide Lecca, Marta Fumagalli

**Affiliations:** Department of Phamacological and Biomolecular Sciences, Università degli Studi di Milano, Via Balzaretti, 9-20133 Milan, Italy; davide.marangon@unimi.it (D.M.); marta.boccazzi@unimi.it (M.B.); davide.lecca@unimi.it (D.L.)

**Keywords:** myelin, lipid metabolism, extracellular matrix, remyelination

## Abstract

Myelin is an essential structure that protects axons, provides metabolic support to neurons and allows fast nerve transmission. Several neurological diseases, such as multiple sclerosis, are characterized by myelin damage, which is responsible of severe functional impairment. Myelin repair requires the timely recruitment of adult oligodendrocyte precursor cells (OPCs) at the lesion sites, their differentiation and maturation into myelinating oligodendrocytes. As a consequence, OPCs undergo profound changes in their morphology, functions, and interactions with other cells and extracellular environment, thus requiring the reorganization of both their lipid metabolism and their membrane composition, which is substantially different compared to other plasma membranes. Despite the growing knowledge in oligodendroglia biology and in the mechanisms involved in OPC-mediated regeneration, the identification of strategies to promote remyelination still remains a challenge. Here, we describe how altered lipid metabolism in oligodendrocytes influences the pathogenesis of demyelination, and we show that several FDA-approved drugs with a previously unknown remyelination potential do act on cholesterol and lipid biosynthetic pathways. Since the interplay between myelin lipids and axons is strictly coordinated by the extracellular matrix (ECM), we also discuss the role of different ECM components, and report the last findings on new ECM-modifiers able to foster endogenous remyelination.

## 1. Introduction

Myelin is the lipid-rich protective covering formed by oligodendrocytes (OLs) that surrounds and protects axons. In their distal portion, the processes of myelinating OLs become large sheaths that wrap axons in a multilamellar fashion to provide insulation and to allow a correct saltatory conduction. Myelin is a compact and dynamic structure spatially organized in highly heterogeneous functional domains, which also gives metabolic support to neurons [1]. To be efficient in their functions, myelin membranes have an extremely high lipid to protein ratio and a different lipid composition compared to typical plasma-membranes [2,3]. In particular, lipids account for about 70% of the myelin membrane, among which cholesterol and glycosphingolipids (i.e., galactosylceramides, sulfatides, gangliosides) are the major components (40% and 20% of the total lipids, respectively). A high amount of cholesterol is required for compaction, whereas glycosphingolipids are necessary to form particular lipid rafts, and their sugar residues are necessary for cell-to-cell communication and interaction with extracellular matrix (ECM) [4].

Damage to myelin sheath is present in different severe neurological conditions such as multiple sclerosis (MS), brain ischemia, and amyotrophic lateral sclerosis (ALS). Loss of myelin ultimately results in reduction of nerve conduction velocity and in altered transfer of energy metabolites to neurons which contribute to disease [5,6]. Myelin repair, through the activation, recruitment and differentiation of adult oligodendrocyte precursor cells (OPCs), which become new myelin forming OLs [7], is crucial for limiting axon degeneration and progressive disease disability. During the remyelination process, OPCs undergo profound morphological and functional changes and progressively remodel their membrane composition, increasing the biosynthesis of cholesterol and galactosphingolipids, and reducing the relative amount of phospholipids and proteins [4]. An intricate interaction of environmental signals and cell-intrinsic mechanisms triggered by the immune and inflammatory response to injury is known to limit the regenerative potential of OPCs in MS [8,9]. However, the role of modulators of lipid metabolism in OPC-mediated repair is still not completely elucidated. Of note, recent studies suggest that targeting the lipid pathways in OLs may be a good strategy to promote remyelination [10].

Furthermore, in MS, remyelination failure is also strictly correlated to an altered extracellular signaling microenvironment that, among others, affects the organization of OL membranes, which causes defects in myelin at the molecular level [11,12]. Although the ECM is one of the main elements that constitute the central nervous system (CNS) parenchyma, new roles for the ECM components in regeneration and repair responses to CNS injury have only recently been documented. Indeed, CNS ECM has emerged as an information-rich environment that can influence cell proliferation, differentiation, migration, synapse formation and remodeling, and responses to injury through the transmission of intracellular signals [13]. Highly relevant, recent studies highlight a link between ECM mechanical cues and alteration of lipid metabolism.

Here, we describe crucial regulators and enzymes involved in lipid biosynthetic pathways showing their potential involvement as targets to promote remyelination. We highlight that different small molecules, some of which already under investigation in clinical trials, have unexpected pro-remyelinating effects acting on enzymes involved in the synthesis of cholesterol or fatty acids (FAs). Finally, we also report recent findings that shed light on the mechanisms by which ECM regulate OL maturation, myelination and remyelination.

## 2. Lipids as Main Components of Myelin Membranes

During their differentiation, OLs undergo a progressive reorganization in lipid metabolism, triggered by changes in gene expression of crucial regulators such as the sterol regulatory element-binding protein (SREBP) and the liver X receptor (LXR). These transcription factors orchestrate the expression and the activity of enzymes involved in cholesterol, FA and triglyceride synthesis [14,15]. In the following paragraphs we will briefly describe the biosynthetic pathways of cholesterol and sphingolipids that represent major components of myelin membranes. Alterations in their correct spatial and temporal expression, transport and degradation may be at the basis of the pathogenesis of demyelination.

### 2.1. Cholesterol Biosynthesis, Transport and Catabolism

Myelin membranes require a large amount of cholesterol, which in physiological conditions is locally produced by nerve cells. De novo cholesterol synthesis is initiated by the 3-hydroxy-3-methylglutaryl-coenzyme-A (HMG-CoA) reductase that converts HMG-CoA into mevalonate. In the three subsequent steps, this intermediate is converted into isopentenyl pyrophosphate, and then, through progressive condensation reactions, to farnesyl pyrophosphate and squalene. This compound cyclizes to form the first sterol, namely lanosterol. Due to its low solubility, lanosterol is handled by a number of membrane enzymes, among which lanosterol 14α-demethylase (CYP51) and Δ8-Δ7 sterol isomerase (also called Emopamil-Binding Protein, EBP), and is finally converted to cholesterol (for review, see [16]).

During development, the main cholesterol producers in the CNS are neurons and OLs, whereas in the adulthood, this function is provided by astrocytes, which regulate both its recycling and transport to neurons and OLs through apolipoproteins. Moreover, cholesterol transport and clearance play a pivotal role in maintaining the correct myelin function.

The major elimination pathway of brain cholesterol is the conversion into 24(S)-hydroxycholesterol (24-OHC), which crosses the blood–brain barrier (BBB), enters the circulation, and is eliminated by the liver. This conversion is carried out by the cholesterol 24-hydroxylase (CYP46A1), an enzyme belonging to the cytochrome P450 family mainly expressed by neurons [17]. During experimental autoimmune encephalomyelitis (EAE), a widely used model of MS, CYP46A1 induction has been described in activated astrocytes, microglia and macrophages inside the demyelination areas. In the same areas, lipids and lipoproteins generated by myelin disruption were clearly visible in the acute phase of the disease, but they completely disappeared in the remission phase. This suggests that CYP46A1 upregulation inside the lesions during the recovery period may have beneficial effects contributing to efficient clearance of myelin debris [18]. The importance of this mechanism has been demonstrated in cuprizone-treated aged mice, in which, on the contrary, myelin-derived cholesterol crystals are not efficiently removed. These debris engulf phagocytes and induce a maladaptive inflammatory response that impairs endogenous remyelination. In these mice, the administration of the LXR agonist GW3965 improved lesion recovery by inducing the expression of genes involved in lipid efflux, such as Abca1, Abcg1, and ApoE [19]. In this respect, drugs being developed to reduce cholesterol levels such as statins may also be good candidates for regenerative medicine in the CNS (see also Section 3.5).

Moreover, during remyelination, the efficient removal of accumulated lipids is accompanied by an increased cholesterol synthesis. Indeed, high-throughput sequencing on single cells revealed that, in the remyelination phase after cuprizone-induced demyelination, the strong downregulation of neuroinflammatory pathways was paralleled by a significant upregulation of the cholesterol synthesis pathways, including important enzymes such as HMGCoA synthase 1 (HMGCS), farnesyl diphosphate synthase (Fdps) and farnesyl-diphosphate farnesyltransferase 1 (Fdft1) [20].

Lipid composition of myelin can change during the course of demyelinating diseases such as MS and leukodistrophies, and this may eventually lead to myelin destabilization or breakdown. During the active phase of MS, an increase in circulating lipids, in particular total cholesterol, has been associated with disability [21]. It is unclear whether high serum cholesterol is a consequence of MS or contributes to disease progression. It is also possible that the peripheral recruitment of cholesterol is a response to counteract local demyelination.

### 2.2. Biosynthesis of Fatty Acids and Sphingolipids

**Figure 1:**
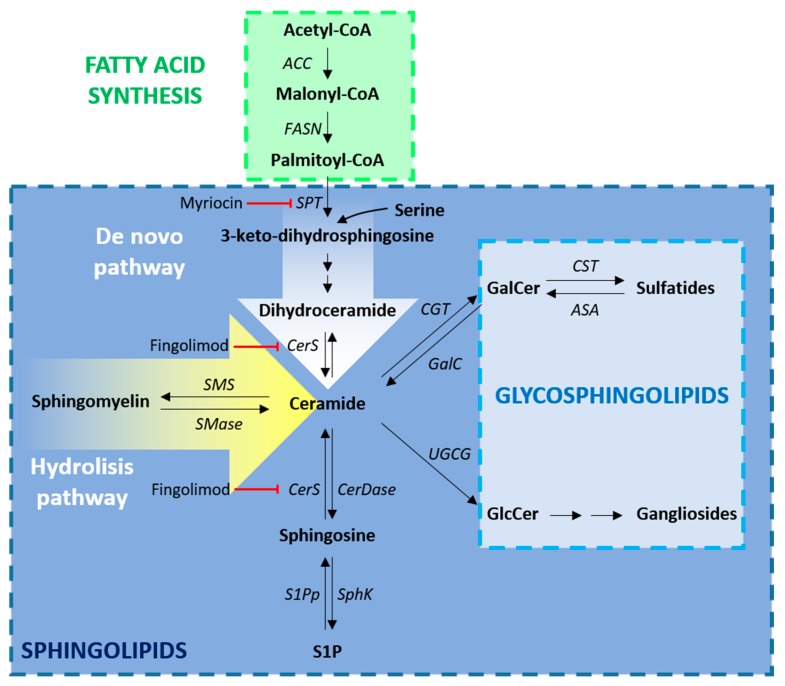
Lipid biosynthesis. Schematic overview of the pathways involved in the synthesis of fatty acids (FAs), sphingolipids and glycosphingolipids. For FA biosynthesis (green box), acetyl-CoA is converted into malonyl-CoA by ACC. The repeated condensation of acetyl-CoA and malonyl-CoA by the multifunctional enzyme FASN leads to the generation of palmitic acid, a fully saturated 16-carbon FA. The synthesis of ceramides (blue box) can occur via 2 distinct pathways: the de novo pathway (white arrow), that begins with the transamination of palmitoyl-CoA by SPT via a condensation reaction with serine and the hydrolysis pathway (yellow arrow), that synthesizes ceramide utilizing sphingomyelins as substrates through the action of SMase. Furthermore, ceramides can be obtained from sphingosine, through the action of CerS, and from GalCer, through GalC. Ceramides serve as precursor for the biosynthesis of the glycosphingolipids (light blu box), sphingomyelin and sphingosine. Enzyme abbreviations: ACC, acetyl-CoA carboxylase; ASA, arylsulfatase A; CGT, UDP–galactose:ceramide galactosyltransferase; CST, cerebroside sulfotransferase; CerS, ceramide synthase; CerDase, ceramidase; FASN, fatty acid synthase; GalC, galactosylceramidase; S1Pp, S1P phosphatase; SMase, sphingomyelinase; SMS, sphingomyelin synthase; Sphk, sphingosine kinase; SPT, serine palmitoyl transferase; UGCG, UDP-glucose ceramide glycosyltransferase. Metabolite abbreviations: GalCer, galactosylceramide; GlcCer, glucosylceramide; S1P, sphingosine-1-phosphate.

FAs are used for the synthesis of both phospholipids and sphingolipids (Figure 1). They can be both introduced with the diet and endogenously synthesized. Endogenous FA synthesis is required for both correct CNS myelination in development and is essential for efficient remyelination. The relevance of efficient FA synthesis in OLs has been recently evaluated in a genetic mouse model in which these cells lacked the fatty acid synthase (FASN), a multifunctional enzyme responsible for the synthesis of the long-chain saturated palmitic acid (C16:0) from acetyl-CoA and malonyl-CoA. It has been demonstrated that in the spinal cord, FA synthesis is not required for correct proliferation and differentiation of OPCs, but it becomes critical in the myelination phase. The analysis of this mouse model demonstrated that the major source of myelin lipids are astrocytes. [10] However, when lipid synthesis is inactivated in astrocytes, OLs are able to bypass this deficiency by recruiting dietary lipids for myelin membrane synthesis [22].

Recent studies showed that increased intake of n-3 polyunsaturated FAs (PUFAs) has beneficial effects due to their neuroprotective and anti-inflammatory properties in a variety of neurodegenerative diseases [23]. Nervonic acid is an unsaturated FA (C24:1n-9) essential for the production of sphingomyelin, a necessary component of myelin [24]. In vivo administration of nervonic acid can improve brain development. Lipid profiling in the brain of EAE mice revealed that during acute inflammation, nervonic acid synthesis is inhibited, with a shift of lipid metabolism pathway of common substrates into proinflammatory arachidonic acid production. Although its remyelinating potential has not been evaluated, cultured human OPCs exposed to nervonic acid increased their maturation rate and the synthesis of sphingolipids [25], suggesting that changing the composition of lipid metabolism may be an important and overlooked prerequisite for remyelination.

As already mentioned, FAs are the building blocks of sphingolipids, whose precursor is ceramide. Serine palmitoyl-transferase (SPT) and ceramide synthases (CerS) are key enzymes in the de novo synthesis of ceramide. In the spinal cord of EAE rats, the pathological increase of TNFα and IFNγ stimulated SPT activity and the consequent transient upregulation of ceramides and its direct product sphingosine, leading to OL apoptosis [26]. The toxic effect of these lipid intermediates was confirmed in human cultured OLs and was counteracted by myriocin, a sphingosine analogue acting as potent inhibitor of SPT [27]. The correct balance between ceramide biosynthesis and degradation is of great importance for myelin maintenance. Indeed, the lack of ceramide synthase 2 (CerS2), one of the enzymes catalysing the biosynthesis of ceramide, strongly reduced the amount of sphingolipids with very long chain FA, leading to aberrant alterations in myelin composition and detachment of myelinic lamellae [28]. Of note, fingolimod, a myriocin derivative currently used in the treatment of MS for its immunomodulatory properties, has been demonstrated to inhibit CerS thus increasing sphingosine and dihydroceramide [29]. Fingolimod crosses the BBB and interacts with the sphingosine-1-phosphate (S1P) glial receptors in vivo. The preventive treatment with fingolimod was demonstrated to ameliorate remyelination in several models of MS including the EAE, but its direct effects on remyelination is still debated [30,31].

Even though sphingolipids are not myelin-specific lipids, their enrichment in myelin is much higher than in any other tissue [32]. The most abundant myelin sphingolipids are galactosylceramides (GalCer) and their sulfated forms, namely sulfatides (Sulf), originating from a sphingosine with long-chain highly saturated FAs, in particular 24:0 and 24:1 [24].

In glial cultures, the synthesis of GalCer and Sulf starts at the onset of terminal differentiation and is maintained in both mature and myelinating OLs. Due to their antigenic properties, several antibodies have been raised against these molecules (e.g., GalC, O4) and their presence in the membrane reflects the transition from OPCs to immature OLs [33]. Decreases in myelin Sulf content in the brain have been described as important factors in the disruption of myelin stability and function [34]. Knock-out mice for UDP-galactose ceramide galactosyltransferase (CGT), the enzyme responsible for GalCer synthesis, exhibit tremors and ataxic locomotion and usually die in the first 3 months of age [35]. This neuropathological phenotype is partly due to defective myelin, with reduced thickness and major alterations in the organization of the paranodal junction, where myelin interacts with the axon. Mice lacking the galactosylceramide sulfotransferase (CST) do not synthesize Sulf, showing a thinner but relatively compact myelin. The pathological phenotype is milder compared to CGT knock-out mice, but the alterations in the paranodal regions are similar [36]. These findings suggest that GalCer and their Sulf are not strictly necessary for the synthesis of myelin, but they are required for stabilizing myelin sheaths and for the interaction with the axons. Indeed, due to their chemical properties, they localize at the outer leaflets of the myelin membranes, and their sugar moieties face to each other during axonal wrapping, interact in the so-called “glyco-synapse” and trigger important structural reorganization, such as cytoskeletal disruption, required for obtaining a compact sheath [37]. Together with cholesterol, galactosphingolipids contribute to the formation of lipid rafts, functionally isolated domains in which they regulate the spatial organization and lateral dynamics of myelin proteins such as the myelin basic protein (MBP), the proteolipid protein (PLP), and adhesion molecules [38,39]. It is still not clear if the resulting downstream signaling is directly mediated by galactosphingolipids or by their interaction with myelin proteins. Indeed, MBP, whose membrane localization is influenced by the presence of GalCer-rich microdomains, is the most important cytosolic protein involved in myelin compaction, and can trigger cytoskeletal rearrangements through its binding with actin. Instead, PLP interacts with both the Sulf component of lipid rafts and extracellular matrix proteins such as laminins [39].

Brain Sulf metabolism is regulated by vitamin K. In the cuprizone model, the administration of vitamin K was able to increase significantly the synthesis of brain Sulf in the remyelination phase, but it did not enhance remyelination per se, in line with the role of Sulf as myelin stabilizers [40].

Alterations in spatial and temporal levels of sphingolipids may severely compromise the maintenance of the functional integrity of the nervous system in several neurodegenerative diseases, including MS. Elevation of lipids such as sphingosine and ceramides may be toxic and cause apoptotic death or degeneration of OLs and neurons [27]. Mass spectrometry analysis revealed that, in normal appearing white matter of active-MS patients, there was a shift in the lipid composition, with higher phospholipid and lower sphingolipid content [41], suggesting that metabolic alterations may appear before demyelinating lesions, thus opening unexplored opportunities in early diagnosis of MS from peripheral fluids. A lipidomic profiling approach led to the identification of enriched levels of ceramide C16:0 and C24:0 in the cerebrospinal fluid of patients with MS. In vitro studies on cultured neurons, revealed that these ceramide species were responsible for mitochondrial dysfunction and oxidative axonal damage [42]. A decrease in the tissue levels and a rise in cerebrospinal fluid levels of Sulf and anti-Sulf antibodies were also reported in MS patients [43,44].

Even if the turnover of lipids in myelin is slow, there is a continuous exchange of molecules, which can be also influenced by dietary lipids crossing the BBB. In the adult brain, each myelin component has a specific turnover rate. A clinical study based on empirical measurements of cerebrospinal fluid from healthy subjects identified β-D-galactosylceramide (β-GalC; 24:1) as the most specific myelin degradation product, with an estimated half-life of 413 days [45]. β-GalC kinetics may be a potential biological means to assess ongoing relevant rearrangements as an alternative to magnetic resonance imaging (MRI) techniques. Additional studies in MS patients are needed to validate this method as an outcome measure in clinical trials testing remyelination therapies.

Gangliosides are glucosylceramides with one or more sialic acids in the head group and represent a huge family of sphingolipids, particularly enriched in brain and they account for 10% of the lipid composition in axons. Gangliosides also take part in myelination, even if they are not major components of OL membranes and myelin. Oligodendroglial inactivation of ceramide glucosyltransferase (UGCG), the limiting enzyme in the synthesis of all the gangliosides, resulted in minor changes in the myelin structure, whereas conditional knock-out mice in neurons, apparently normal at birth, showed progressive peripheral demyelination, ataxia, severe motor defects, and died within three weeks [36]. These data suggested that gangliosides do organize myelin behavior through axonal signals. B4galnt1-null mice, which lack all complex gangliosides, display significant axonal degeneration in both CNS and peripheral nervous system, reduced axon caliber and motor behavioral deficits and neuropathy, suggesting that complex gangliosides are essential for the maintenance of axon-myelin long-term stability. Moreover, in the cuprizone-induced demyelination model, the treatment with the ganglioside GD1a increased OPC proliferation and differentiation [46]. Nevertheless, it has also been shown that specific gangliosides, such as GD1a and GT1b, physically interact with the myelin associated glycoprotein (MAG), located at the innermost layer of myelin membranes, and may act as axonal regeneration inhibitor [47]. Accordingly, the treatment with sialidase, an enzyme that cleaves sialic acid from gangliosides, strongly enhanced the regeneration of spinal axons in the rat model of brachial plexus avulsion, a traumatic injury in which nerve roots are torn from the spinal cord [48]. The apparently contrasting role of GD1a on OPC maturation and axonal regeneration may also depend on the accumulation of GM1, the product of sialidase action on major brain gangliosides, which has shown a neuroprotective activity [49] but failed to enhance myelin membrane formation [46]. Therefore, ganglioside-modifying treatments might be administered during a critical period after damage to allow axonal regeneration, but then they should be halted to ensure long term myelin stability.

Taken together, these findings highlight that myelin is not merely an insulating covering, but a highly dynamic environment in which structural lipids also behave as signaling molecules. Remyelinating approaches targeting the biosynthesis of these molecules or their regulation may contribute to establish the correct organization of myelin membranes during remyelination.

## 3. Drugs Promoting Remyelination through a Potential Action on Oligodendrocyte Lipid Metabolism

All the currently available therapies for MS are immunosuppressants and immunomodulators [50]. They do ameliorate the length of relapses and the related symptoms, but their severe side effects and inefficacy in counteracting the long-term outcome of the disease prompted to consider new strategies to recovery/improve neurological functions of patients. In this respect, drug-based therapies able to promote endogenous remyelination capability of OLs are now emerging as major and essential approaches to demyelinating disorders [7]. Interestingly, a consistent number of the most promising remyelinating molecules acts on the synthesis of cholesterol or FAs that, as explained in detail in the previous sections, are emerging as key factors in OL differentiation.

**Figure 2:**
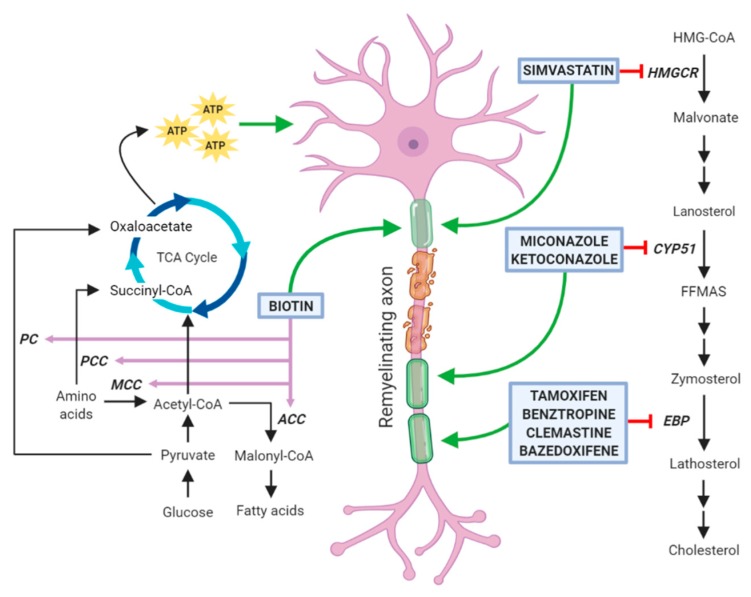
A consistent number of the most promising remyelinating molecules acts on enzymes involved in the biosynthesis of fatty acids or cholesterol. Remyelinating drugs currently under investigation for multiple sclerosis in pre-clinical or clinical trials are reported. Among them biotin can act in oligodendrocytes as co-factor for the enzyme acetyl-CoA carboxylase (ACC) which is the rate-limiting and committing step for the synthesis of malonyl-CoA, the building block for fatty acid synthesis. Biotin can also induce an increased ATP pool in neurons, by acting as co-enzyme for propionyl-CoA carboxylase (PCC), 3-methylcrotonyl-CoA carboxylase (MCC) and pyruvate carboxylase (PC). On the other hand, simvastatin, miconazole, tamoxifen, benztropine, clemastine and bazedoxifene inhibit enzymes located at different levels of the cholesterol biosynthesis. Abbreviations: HMG-CoA, 3-hydroxy-3-methyl-glutaryl-coenzyme A; HMGCR, HMG-CoA reductase; CY51, lanosterol 14α-demethylase; FFMAS, follicular fluid meiosis-activating sterol; EBP, Emopamil-Binding Protein.

Several papers published in recent years have tested libraries of compounds, including already Food and Drug Administration (FDA)-approved drugs in high-throughput screenings and have identified new molecules with previously unknown remyelination potential [51,52,53]. Although some compounds (such as tamoxifen and clobetasol) exert their positive therapeutic effects counteracting the detrimental action of immune cells and cytotoxic glia, a considerable number of these molecules has been also reported to directly enhance OPC differentiation and to share the capability of inhibiting enzymes involved in cholesterol biosynthesis in these cells (Figure 2). However, it cannot be excluded that these agents may also affect lipid biosynthesis on other neural cells. Among them, clemastine, benztropine, and some selective estrogen receptor modulators (SERM) inhibit the enzyme EBP, whereas miconazole and ketoconazole, two imidazole antifungals, act through the inhibition of the enzyme CYP51, all ultimately leading to the accumulation of the two 8,9 unsaturated sterols zymosterol and lanosterol, respectively [54,55]. It is worth to mention that alternative mechanisms of action may contribute to a direct increase of OL differentiation. Indeed, miconazole and ketoconazole appear to activate protein kinases [53,56,57,58]. In particular, miconazole induces a strong phosphorylation of ERK1/2, whose constitutive activation has been correlated with a profound increase in the extent of remyelination after toxin-induced demyelinating injury [53,59]. In addition, clobetasol, an agonist of glucocorticoid receptor widely used to treat different pathological skin conditions [60], can also act through the smoothened (Smo) receptors [61], whose activity is regulated by cholesterol intermediates [57,58]. As reported below, the efficacy of all these drugs in enhancing OL differentiation has been well documented by several publications and, in the case of clemastine, also by two clinical trials on MS patients. Additionally, statins, classic inhibitors of the HMG-CoA reductase, are currently under investigation as pro-remyelinating drugs. However, there are conflicting data from both preclinical and clinical studies regarding their direct role in enhancing OL myelination (see Section 3.5). Finally, we will describe biotin as a promising remyelinating molecule, due to the its role as a cofactor for acetyl-CoA carboxylase (ACC), the rate limiting enzyme in the synthesis of malonyl-CoA, the building block for FA synthesis.

### 3.1. Clemastine

Clemastine fumarate is a widely available first-generation antihistamine with a favorable safety profile, primarily used for symptomatic treatment of seasonal allergies. It was firstly identified as one of the hit compounds with potential remyelinating capability through different high-throughput screening approaches aimed at stimulating differentiation of primary rodent OPCs and pluripotent stem cell-derived OPCs [52]. The positive effect of clemastine on remyelination was also demonstrated in vivo in the lysolecithin (LPC)- and cuprizone-induced demyelination models [52,62] and in a murine model of neonatal hypoxic injury [63,64]. Clemastine was also shown to enhance myelination in the prefrontal cortex of socially isolated mice [65]. These very promising preclinical results led to a phase 2 double-blind, randomized, placebo-controlled, crossover trial (ReBUILD; NCT02040298) aimed at testing the efficacy of clemastine fumarate for remyelination [66]. This study enrolled 50 patients with relapsing-remitting MS (RRMS), mild neurological disability, and chronic optic neuropathy, which have been randomly assigned to receive 5.36 mg oral clemastine fumarate twice daily for 90 days followed by placebo for 60 days or placebo for 90 days followed by clemastine fumarate for 60 days. Clemastine fumarate treatment reduced visual-evoked potential latency (a measure of optic nerve remyelination) accompanied by a trend for improvement in low-contrast letter acuity, the leading outcome measure to assess visual disability in MS. However, several MRI techniques able to measure myelin integrity (myelin water fraction, magnetization transfer imaging, and diffusion tensor imaging) could not identify any beneficial outcome. Clemastine treatment was associated with fatigue but no serious adverse effects have been observed. A second and randomized, double-blind, parallel-group and placebo-controlled clinical trial is still ongoing (ReCOVER, NCT02521311). Patients with acute optic neuritis will be treated with clemastine for 3 months, off treatment from 3–9 months and reevaluated at 9 months. Study procedures will include assessments for evidence of remyelination in the anterior visual pathway and in the brain using electrophysiologic techniques and MRI.

### 3.2. Benztropine

Another FDA-approved drug, whose pro-remyelinating effects obtained in preclinical studies were very encouraging, is benztropine. This muscarinic antagonist, currently in use for treatment of Parkinson’s Disease, was highly effective in decreasing clinical severity both in the cuprizone and in the EAE model [51]. In the PLP-induced EAE rodent model of RRMS, benztropine enhanced remyelination both when administered prophylactically starting on the day of PLP immunization or when the drug was administered therapeutically at the first signs of disease. Even though in this model benztropine treatment did not influence the infiltration, activation or proliferation of T cells or other immune effector cells, it has been shown to directly enhance OPC maturation. Moreover, in the EAE model the use of benztropine in combination with INFβ or fingolimod, two approved immunomodulatory drugs, enhanced functional recovery and, in the case of fingolimod, allowed to significantly decrease the dosages of both drugs to achieve equivalent efficacy [51]. However, no clinical trials are currently ongoing on benztropine.

### 3.3. Selective Estrogen Receptor Modulators

Tamoxifen and other selective estrogen receptor modulators (SERMs) have become promising therapeutic targets for demyelinating diseases due to their potential contribution in differentiation of OLs. Tamoxifen belongs to the triphenylethylene family of nonsteroidal SERM and is already used to prevent or treat breast cancer [67]. Thus, its repurposing as remyelinating drug may have several advantages compared to the development of entirely new compounds since its safety profile has been confirmed by exhaustive clinical applications. Tamoxifen promoted OPC differentiation in vitro by acting on ERα, ERβ or GPR30. Tamoxifen treatment was also able to stimulate remyelination in vivo, in two different models of focal demyelination, i.e., injection of lysolechitin in the corpus callosum or of ethidium bromide into the WM tracts of the rat brainstem [54,68]. Besides targeting OLs, tamoxifen has also been reported to counteract in vivo the activation of microglia and astrocytes in models of neuroinflammation [69], likely contributing to create a permissive environment for efficient remyelination. Bazedoxifene (BZA), another FDA-approved SERM, was described as a potent agent able to enhance the differentiation of human embryonic stem cell derived-OPCs into mature MBP+ cells. Moreover, BZA-treated OPC/dorsal root ganglia co-cultures showed significantly more myelin internodes, proving that BZA is also able to enhance myelination of axonal substrates. In vivo, BZA increased and accelerated remyelination in the lysolecithin model. Interestingly, these positive effects were maintained when the experiment was performed in ERα-ERβ double knock-out mice, suggesting that BZA act through an ER-independent mechanism. Indeed, a sophisticated computational analysis identified EBP as one out of the six possible candidate targets through which BZA and other SERMs, such as tamoxifen, could promote remyelination [55]. The strong pre-clinical evidence for the remyelinating potential of BZA prompted a phase 2 randomized, placebo controlled, double-blind, delayed-start trial, now at the initial stage (ReWRAP, NCT04002934). The primary goal of this study is to assess the efficacy of this compound as remyelinating agent in patients with RRMS. The investigators will utilize electrophysiologic techniques and MRI to longitudinally assess the pro-myelinating effect of BZA in 50 women over the course of 6 months.

### 3.4. Clobetasol and Miconazole

Both these drugs have been identified as the two most active remyelinating drugs in high-throughput screenings aimed to test a library of bioactive small molecules on mouse pluripotent epiblast stem cell-derived OPCs [53]. Both compounds were also effective in promoting precocious myelination in organotypic cerebellar slice cultures, and in early postnatal mice. Moreover, systemic delivery of each of the two drugs significantly increased remyelination in the LPC model, and ameliorated disease severity when administered in MOG_35–55_-induced EAE, which mimics chronic inflammatory demyelination [53]. To interpret the potential impact of the two drugs as therapeutics in immune-mediated MS models, their effects on immune cells survival and function have been also tested. Interestingly, only clobetasol altered naïve T cell differentiation, proliferation and secretion of cytokines from lymphocytes in PLP_139–151_-induced EAE mice (an immune-driven RRMS model), demonstrating that miconazole functions directly as a remyelinating drug with no effect on the immune system, whereas clobetasol is also a potent immunosuppressant [53].

### 3.5. Simvastatin

As mentioned above, statins, a class of drugs commonly used for the treatment of hypercholesterolemia, inhibit the HMG-CoA reductase, an upstream enzyme in the cholesterol biosynthesis pathway. Due to initial encouraging results obtained in pre-clinical studies, several clinical trials exploiting the possibility to use simvastatin for MS have been conducted or are still ongoing. However, it should be taken into account that long-term statin therapy might promote cerebral small vessel disease and impair myelination, perhaps resulting from cholesterol depletion and pleiotropic effects on amyloid-b metabolism and OL function [70].

A number of studies have suggested that statins may ameliorate the signs and symptoms of acute inflammatory demyelinating disease by modulating the neuroinflammatory response (reviewed in [71] and [72]). In addition, complementary studies have focused on the effects of statins on OL differentiation and myelin production. Conflicting data from both preclinical and clinical studies emerged on the role of simvastatin in OL differentiation and remyelination. Prolonged exposure to simvastatin of mature OLs was associated with process retraction (6 days) and increased cell death in vitro (eight days; [73]). In agreement, the in vivo intraperitoneal injection of simvastatin significantly inhibited the spontaneous remyelination that occurs after the cuprizone-induced demyelination [74].In contrast, glial progenitor cells treated with statins are more committed towards the OL lineage [75] and short-term treatment (1 day) of human and rodent OPCs induced process outgrowth and differentiation [73]. Moreover, the beneficial action of simvastatin in reducing initial disease severity in the EAE model of MS [76] has encouraged to move ahead in the clinical trials.

The reported conflicting results on the efficacy of simvastatin for MS treatment, and more in general of statins, could be explained by intrinsic differences between in vitro studies and the in vivo models used for the experiments and by the complex and not yet fully elucidated mechanism of action of these drugs. Indeed, in vitro experiments cannot reproduce the complex interaction between neural and immune cells, which characterized the disease. Finally profound differences also exist between cuprizone and EAE, being the former an in vivo model of toxin-induced demyelination with spontaneous remyelination and the latter a model of chronic inflammatory autoimmune demyelination [77].

Regarding clinical trials, positive results were obtained in an open-label, single-blind, crossover study in which 30 patients with RRMS were treated with simvastatin 80 mg daily for 6 months and showed a reduction in the number and volume of gadolinium-enhancing lesions. However, in a larger (380 patients) randomized trial investigating whether simvastatin could be used as an add-on therapy to IFN-b in RRMS, no beneficial effects were observed using the annual relapse rate as primary endpoint (NCT00492765, SIMCOMBIN; [78]). Nevertheless, in a subsequent randomized, double-blind, placebo-controlled phase II trial (MS-STAT), enrolling 140 progressive MS (PMS) patients, simvastatin (80 mg) led to a significant reduction in the rate of brain atrophy [79] compared to placebo. In a secondary analysis of the same trials aimed at analyzing the treatment effect on cognitive, neuropsychiatric, and health-related quality-of-life outcome measures, a positive effect of simvastatin on frontal lobe function and a physical quality-of-life measure were reported [80]. Based on this result, a phase 3 and multicenter study (MS-STAT2, NCT03387670) is now in the recruiting phase. It will enroll 1180 PMS patients and will investigate the potential impact of simvastatin on disease progression over a period of three years.

### 3.6. Biotin

Two mechanisms have been hypothesized as the basis of the therapeutic effects of biotin in the CNS. The former is linked to the role of biotin as co-factor for the enzyme acetyl-CoA carboxylase (ACC), which is the rate limiting and committing step for the synthesis of malonyl-CoA. The latter is linked to the involvement of biotin as a co-enzyme for propionyl-CoA carboxylase (PCC), 3-methylcrotonyl-CoA carboxylase (MCC), and pyruvate carboxylase (PC), three carboxylases involved in the metabolism of pyruvate and amino acids. These three enzymes are expressed by neurons and their actions lead to an increase in the intraneuronal pool of ATP which in turn can have a neuroprotective effect by reducing the dysfunctions of damaged neurons [81].

MD1003 (MedDay Pharmaceuticals, Paris, France) is a high-dose (300 mg) oral formulation of biotin with hypothesized remyelinating and neuroprotecting potential. A first indication for MD1003 efficacy came from an uncontrolled-non blinded proof of concept study in which 23 patients with PMS were enrolled and treated with 100–300 mg/day of biotin for 2 to 36 months [82]. Overall, the 91.3% of patients exhibited clinical improvement with high dose of biotin. In particular, four patients with chronic visual loss related to involvement of optic nerves exhibited improvement of visual acuity whereas 18 patients with prominent involvement of the spinal cord (11 tetraparesis and 7 paraparesis) displayed clinical improvement evaluated using walking distance, expanded disability status scale (EDSS), TW25 (time to walk 25 feet) muscle strength testing and videotaped clinical examination in a subset of patients. The efficacy of MD1003 was further assessed in two multi-centric double-blind placebo controlled trials [83,84]. The MS-SPI study (NCT02220933; [84]) involved 154 patients and consisted in a 12-month randomized, double-blind, placebo-controlled trial followed by an open-label 12-month extension phase where all patients received MD1003 (100 mg thrice daily). 12.6% of patients treated with MD1003 reached the primary end point of the study i.e., the reduction of MS-disability measured by an EDSS decrease of ≥1 or a ≥20% decrease in TW25 at 9 months and 12 months, compared to none in the placebo arm. Moreover, MD1003 treatment also reduced EDSS progression and improved clinical impression of change compared with placebo.

The MS-ON study (NCT02220244; [83]) was a six month randomized, double-blind, placebo-controlled trial with a six-month open label extension phase which involved 93 patients suffering from chronic visual loss after acute optic neuritis or slowly progressive optic neuropathy related to MS. Although treatment did not significantly improve visual acuity compared with placebo, a trend favoring MD1003 was observed in the subgroup of patients with progressive optic neuropathy.

A further phase 3 clinical trial (NCT02936037; SPI2) is currently underway to demonstrate the superiority of MD1003 over placebo in counteracting disability of patients suffering from PMS and especially those with gait impairment. In this trial, 90 clinical study centers are active worldwide (48 centers in the United States and Canada, 39 centers in Europe and 3 centers in Australia) and the estimated Study Completion Date is June 2023.

## 4. Essential Roles of ECM for Timely Development of OPCs into Myelinating Oligodendrocytes

In the CNS, ECM is composed of proteoglycans, hyaluronan and multiple protein components such as fibronectin and laminin [85]. Of great interest, the regulation and transient expression of these distinct ECM molecules is essential in maintaining the proper physiological environment for timely development of OPCs into myelinating OLs [86]. Indeed, as observed in 70% of MS lesions, unbalanced expression of these ECM entities, mainly produced by astrocytes, contributes to the formation of a non-permissive barrier at the lesion edge, blocking OPC migration and differentiation, and the subsequent remyelination process [87]. ECM proteins may also act directly on other neural cells, such as astrocytes, and potently modify their functions. In vitro studies have shown that astrocytes behavior and their molecular signature strictly depend from the ECM proteins on which they are grown, suggesting that astrocyte activation is an ECM substrate-dependent phenomenon [88].

**Figure 3:**
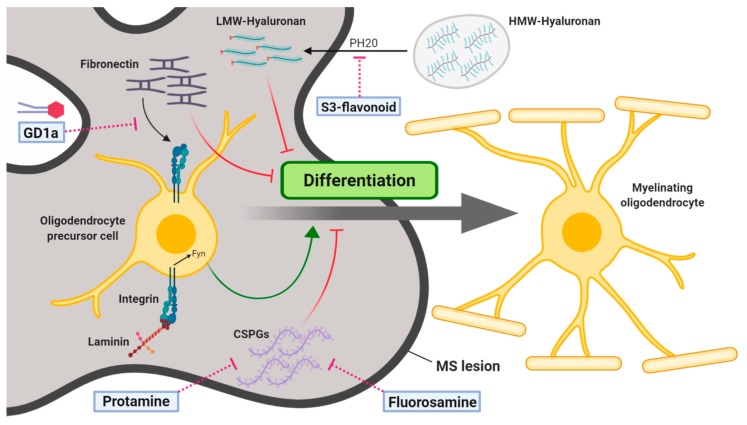
Targeting of extracellular matrix components to foster oligodendrocyte maturation and remyelination. In MS lesions, an unbalance expression of extracellular matrix components (fibronectin, hyaluronan, CSPGs) contributes to form a non-permissive barrier at the lesion edge, blocking the migration of oligodendrocyte precursor cells (OPCs), their maturation and the subsequent remyelination process. The differentiation of OPCs to myelinating oligodendrocytes can be enhanced by pharmacological agents (GD1a, S3-flavonoid, Protamine, Fluorosamine) that counteract the synthesis and accumulation of these factors. MS, multiple sclerosis, LMW, low molecular weight; HMW, high molecular weight; CSPGs, chondroitin sulfate proteoglycans.

Interestingly, ECM undergoes extensive remodeling following CNS injury during both development and adulthood (e.g., perinatal hypoxic-ischemic cerebral injury, age-related vascular dementia, MS) [89]. Although attempts to restore their activity through pharmacological intervention represent another promising therapeutic strategy for demyelination (Figure 3), as far as we know, none of the ECM components is targeted in the current clinical trials evaluating remyelination in MS.

Very recently, two independent studies have linked the alteration of lipid metabolism to ECM mechanical cues, showing that the physical cell microenvironment modulates the activation of SREBP by affecting translocation of SREBP Cleavage-Activating Protein (SCAP) from the endoplasmic reticulum to the Golgi apparatus and in turn driving lipid synthesis and accumulation [90,91]. These studies also suggest that physiological or pathological conditions leading to altered tissue stiffness may affect SREBP activity and lipid metabolism. In line with this hypothesis, altered SREBP1 expression is present in human samples datasets of normal breast tissue with high vs. low mammographic density, and in idiopathic pulmonary fibrosis patients vs. healthy controls [90]. In MS this homoeostatic interplay might be overcome at multiple levels. Indeed, excessive deposition of ECM constituents, which is typical of active demyelinating MS lesions [85], may directly affect lipid and cholesterol synthesis and, in turn, impede myelin repair.

### 4.1. Chondroitin Sulfate Proteoglycans

Chondroitin sulfate proteoglycans (CSPGs) are proteoglycans consisting of a central core protein with varying number of associated glycosaminoglycan (GAG) chains and a distinct tetrasaccharide linker that mediates the interaction between the core protein and GAGs. CSPGs are a class of ECM components widely expressed within the CNS that can be synthesized by all neural cell types [92]. CSPG functions depend on the core protein, number of chondroitin sulfate GAGs, and sulfation patterns of GAG chains. CSPG family members can be distinguished by their core proteins and they include the four lecticans (i.e., aggrecan, brevican, neurocan, and versican), RPTP/phosphacan, and neuron-glial antigen-2 [93]. It is known that CSPGs are strongly upregulated in response to white matter injury by OPCs, astrocytes and microglia. Several in vitro studies have explored the effects of CSPGs on OPC development, showing that CSPGs did not alter OPC proliferation [94], but impair OPC morphological differentiation and myelin deposition [95,96]. Interestingly, this effect was counteracted by the presence of laminin, indicating that CSPGs might competitively inhibit the β1-integrin signaling pathway during OPC maturation [94]. Moreover, an impaired adhesion and process outgrowth was found when neonatal or adult mouse OPCs were plated on CSPGs. To overcome the inhibitory nature of CSPGs on OPC growth, Keough and colleagues treated OPC cultures with different agents that were recently identified as promoter of OPC differentiation in vitro and remyelination in vivo (see Section 3). Among the tested drugs, benztropine and clemastine (already mentioned above) were the only compounds able to induce a small but significant increase in process outgrowth in presence of CSPGs [97]. In the same study, the authors took advantage of fluorinated N-acetylglucosamine analogues, such as fluorosamine, to reduce the production of CSPGs by targeting different stages of its biosynthesis. Treatment of astrocytes with flurosamine reduced the production of CSPGs and, consequently, partial rescued OPC outgrowth on astrocyte ECM (enriched in CSPGs).

Moreover, in vivo treatment with fluorinated glucosamine derivatives accelerates the rate of OL maturation and remyelination in vivo in the LPC model and significantly reduced EAE clinical severity [98]. Interestingly, some of these compounds, such as difluorosamine (Ac-4,4-diF-GlcNAc), are also able to reduce splenocytes proliferation in vitro and to decrease monocytes and lymphocytes infiltration into the spinal cord of EAE mice if administered prophylactically. Another compound that exhibited a pro-differentiative effect on OPCs in presence of CSPG components is protamine, a polycationic peptide that is widely used clinically to stop the anticoagulant effects of heparin and that showed a high affinity for aggrecan-coated substrates. Intranasal administration of protamine enhanced OL differentiation in the developing mouse brain and remyelination in cuprizone mouse model [99].

Despite these promising data, another study has shown that manipulating proteoglycans in MS could also lead to deleterious effects. Indeed, some CSPGs (such as versican) are well known to inhibit remyelination, but others (such as aggrecan) may also be essential as protective factors during inflammation. In this respect, surfen, a compound known to bind the GAG side chains of proteoglycans with high affinity, has shown a protective effect in the EAE model, but a detrimental effect in the LPC model [100], probably due to a generalized increase in CSPG expression.

### 4.2. Hyaluronan

Hyaluronan (HA) is a negatively charged glycosaminoglycan polymer implicated in the regulation of neural differentiation, survival, proliferation, migration, and cell signaling in the CNS. High levels of HA were found in the white matter lesions of premature infants, adult stroke, MS, traumatic spinal cord injury, and vanishing white matter disease [101]. This accumulation was associated to increased astrogliosis and accumulation of OPCs that fail to reach the terminal maturation [102]. Thus, astrocytic dysfunction may contribute to the consequences of inflammatory HA accumulation in white matter pathologies. HA interacts with CD44 and TLR2/4 receptors, which are predominantly expressed on astrocytes and OPCs. Conditional overexpression of CD44 in the oligodendroglial lineage caused widespread CNS dysmyelination and progressive demyelination [103]. Moreover, CD44 levels were found chronically elevated in demyelinating lesions of multiple sclerosis patients [104]. OPCs express multiple hyaluronidases (HYAL1, HYAL2, and PH20) and can digest HA by producing fragments of low molecular weight (LMW) and high molecular weight (HMW), which present a wide range of biological functions [102]. It has been shown that HA digestion by PH20 (but not other hyaluronidases) inhibits OPC maturation into mature OLs and impairs remyelination following LPC-induced demyelination. In contrast, inhibition of PH20 activity leads to increased OPC maturation and promotes conduction velocities through lesions [105]. The most potent and best characterized pharmacological inhibitors of hyaluronidase activity is Vcpal (L-Ascorbyl 6-palmitate). Despite Vcpal treatment is able to promote in vitro OPC maturation and in vivo remyelination, the needed very high concentration can also lead to inhibition of lipoxygenases [105]. Very recently, Su and colleagues developed a modified flavonoid (called S3) that potently and selectively inhibits the enzymatic activity of PH20 but not Hyal1 or Hyal2. S3 treatment was able to improve OPC maturation in vitro in presence of HMW HA and to promote functional remyelination in the LPC-induced demyelination model [106], suggesting that selective hyaluronidase inhibitors can be exploited to foster endogenous remyelination following CNS injuries. Several flavonoids (i.e., apigenin and hesperidin) have been shown to exert neuroprotective and immunomodulatory activity in the EAE model [107,108], suggesting that flavonoids can be potentially used as therapeutic compounds to counteract dysfunctional processes causing demyelination.

### 4.3. Fibronectin

Fibronectin (Fn) is a high-molecular weight, insoluble glycoprotein dimer that consists of three types of repeating amino acid modules (named type I, II, and III). It has been shown that the Fn matrix is essential for normal embryonic development, whereas, in healthy adult tissues, Fn is expressed at very low levels. In the CNS, Fn re-expression is a transient common response to tissue injury, particularly after myelin damage. Despite the transient expression of dimeric Fn represents a physiologically relevant factor associated to OPC proliferation following CNS demyelination [109], its persistent expression is responsible of remyelination failure. Accumulation of Fn aggregates was indeed found in the parenchyma of active demyelinating MS lesions, suggesting that fibronectin may be locally produced by glial cells and infiltrated macrophages in the CNS [110]. Interestingly, inhibition of OPC maturation was observed when OPCs are cultured in presence of astrocyte-derived matrix that contains fibronectin aggregates, but not in presence of dimeric fibronectin. Moreover, intralesional injection of Fn aggregates in the LPC model perturbed OLs differentiation in vivo and impaired remyelination [12]. These findings prompted for the development of 2 different therapeutic approaches based on the application of Fn aggregate antagonists or the fostering of Fn aggregates clearance from the lesions. A first strategy took advantage of gangliosides, cell surface glycosphingolipids (see also Section 2.2) known to interfere with the interaction between Fn and integrins, their cell surface receptors. Exogenous addition of ganglioside GD1a antagonized the inhibitory effect of aggregated Fn on myelin membrane formation in primary OLs and in the cuprizone-induced demyelination model [46]. The underlying mechanism appears related to a specific GD1a-dependent activation of a PKA signaling pathway, which reflects an increased OPC proliferation and maturation in the lesioned area. About the second approach, that aims to target fibronectin clearance, it has been demonstrated that matrix metalloproteinase-7 (MMP7), a protease involved in ECM remodeling which is typically down-regulated in MS active and chronic lesions, is able to cleave aggregated fibronectin enhancing its clearance [86].

A recent paper described the functional significance of fibronectin accumulation in MS investigating the temporospatial profile of fibrotic scar formation and its contribution to OL differentiation in EAE. This study showed that at the acute and chronic phases of EAE, 70%–85% of fibrotic areas were associated with fibronectin deposition and 60%–70% of the fibronectin positive area was associated with fibroblasts, which play a pivotal role in the alteration of the injury environment through the production of ECM molecules. Moreover, they established that fibroblasts actively inhibit the differentiation of OPCs into mature OLs through the production of inhibitory soluble factors (such as fibronectin) and/or ECM components [111].

### 4.4. Laminin

Laminins are major components of the basal lamina and are also present in the ventricular zone (VZ) of the developing neocortex [112]. Several studies have explored the role of laminin-2 (Lm2, also known as merosin) in the regulation of OL development and CNS myelination. The interaction of Lm2 with the oligodendroglial receptor α6β1 integrin is able to promote OPC maturation. On the contrary, block of this interaction leads to impaired OPC maturation and production of myelin sheaths, which result smaller [113]. Laminin-integrin interactions are also involved in the regulation of Fyn-mediated process dynamics in differentiating OPCs, affecting both process length and branching. The mechanism of action involves the recruitment of receptor protein tyrosine phosphatase (PTP) alpha, which, by forming a complex with α6β1 integrin and the major nonintegrin ECM receptor dystroglycan, transduce Fyn activation upon Lm2 engagement [114]. A recent study also showed that the positive effect of Lm2 on OPC maturation is influenced by substrate stiffness, suggesting that the presence of compliance substrate is determinant for the full differentiation of OLs [115]. In human, Lm2 deficiency, caused by mutations in the LAMA2 gene, is responsible for the development of a congenital muscular dystrophy that is characterized by developmental abnormalities in brain myelination, consequence to a reduced number of mature OLs [116]. White matter abnormalities were also found in Duchenne muscular dystrophy (DMD), a pathological condition caused by mutations in the gene that encodes dystrophin, a protein that connects the ECM to the cytoskeleton. In this respect, a recent study showed that dystrophin loss in OLs delayed their maturation and myelination, suggesting that the loss of oligodendroglial dystrophins contributes to DMD-related neurological deficits [117].

In addition to its ability to bind to dystroglycan and integrin, Lm2 also displays binding affinity toward sulfatide. Interestingly, a recent study provided evidence that Lm2-mediated differentiation of OPCs, and therefore myelination, is dependent on sulfatide. Anti-sulfatide antibodies disrupted integrin α6-PDGFRα interactions on laminin-2 and induced demyelination in myelinated spheroid cultures, but intriguingly stimulated myelin-like membrane formation on fibronectin [118]. Thus, given the accumulation of fibronectin aggregates inside lesions of MS patients [12], the use of modified-anti-sulfatide antibodies may represent a promising therapeutic tool to foster endogenous remyelination.

## 5. Conclusions

During development, OPCs undergo all the morphological and gene expression changes necessary for their differentiation and maturation into myelinating OLs. Developing OPCs progressively reorganize their lipid metabolism and change their membrane composition, greatly increasing the biosynthesis of cholesterol and galactosphingolipids and reducing the relative amounts of phospholipids and proteins. In the adult CNS, when physiological myelination is completed, mature oligodendrocytes continuously replace their myelin components with new lipids coming from dietary intake or through de novo synthesis. Myelin lipid metabolism is sustained by astrocytes, which represent the major source of cholesterol and FAs in the CNS, thus contributing to myelin maintenance and turnover.

During demyelinating diseases, such as MS, myelin is destabilized and disrupted. Due to its very complex and highly polarized architecture its repair is very challenging. On one hand, microglia and infiltrating macrophages clear myelin debris from the extracellular environment, but the large amount of cholesterol may engulf phagocytes, thus reducing their beneficial capabilities. At the same time, they produce cytokines that sustain local inflammation, together with astrocytes, whose trophic role for myelin and neurons is compromised. Oligodendrocytes cannot face the whole energy requirement for myelin maintenance and their lipid biosynthesis is not sufficient for remyelination. Resident OPCs are recruited at the lesion sites, proliferate and differentiate, but often they cannot become mature due to the presence of a highly inflammatory environment. Moreover, in response to lesions, virtually all the glial cells overproduce ECM components, in particular fibronectin and proteoglycans, whose inhibitory effect on remyelination and axonal regeneration is well documented [13]. Of note, ECM mechanical cues induce alteration in the activity of SREBP, a master regulator of lipid metabolism activated by the presence of sterol intermediates.

In this review, based on the recent literature, we have speculated that approaches targeting either lipid metabolism or ECM synthesis may contribute to restore a correct organization of myelin membranes and promote remyelination.

Several studies have associated de novo FA synthesis in maintaining survival of newly mature OLs, suggesting a potential implication of this pathway in the development of efficient remyelination strategies. In accordance, high-dose biotin, currently under investigation in clinical trials for MS, acts as co-factor for the acetyl-CoA carboxylase, the rate-limiting enzyme in the synthesis of malonyl-CoA, the building block for FA synthesis. The amelioration of myelin composition may be also potentially achieved with a diet rich in unsaturated FAs.

Moreover, also enzymes involved in the biosynthesis of glycosphingolipids emerged as putative targets to enhance myelin repair and reduce axonal injury. However, it is worth to note that fostering or inhibiting brain lipid metabolism may unbalance the ratio between lipid intermediates, such as ceramide and sphingosine, which may eventually become toxic for myelin and axons.

The pharmacological inhibition of early enzymes involved in the cholesterol biosynthesis that prevents the synthesis of all cellular sterols, for example using statins, has produced conflicting results on myelin repair. However, several small molecules, including some FDA-approved drugs, may have pro-myelinating activity through the inhibition of specific enzymes in the cholesterol biosynthesis pathway and to the consequent accumulation of 8,9-unsaturated sterol intermediates that signal to enhance oligodendrocyte formation.

Finally, we have also highlighted that some potentially promising pharmacological agents may have reduced efficacy due to the non-permissive extracellular environment, and in particular to the accumulation of ECM. For this reason, remyelinating treatments should be aimed both at fostering the endogenous program of OLs and at removing the inhibitory cues coming from the lesions.
